# Inhibition of SIRT2 limits tumour angiogenesis via inactivation of the STAT3/VEGFA signalling pathway

**DOI:** 10.1038/s41419-018-1260-z

**Published:** 2018-12-18

**Authors:** Fuqing Hu, Xuling Sun, Geng Li, Qi Wu, Yaqi Chen, Xi Yang, Xuelai Luo, Junbo Hu, Guihua Wang

**Affiliations:** Department of Gastrointestinal Surgery Center, Tongji Hospital, Tongji Medical College, Huazhong University of Science and Technology, Wuhan, Hubei 430030 China

## Abstract

Mounting evidence has demonstrated that angiogenesis plays an important role in tumour progression. However, the key regulators in tumour angiogenesis remain unclear. Recently, emerging reports have indicated that SIRT2 plays critical roles in proliferation, metastasis and tumourigenesis in diverse tumours. However, the function of SIRT2 in tumour angiogenesis and the mechanism underlying the regulation of angiogenesis by SIRT2 are still unknown. Here, we found that SIRT2 was upregulated in colorectal cancer tissues compared to that in normal samples and that the elevated SIRT2 was associated with poor prognosis in patients with colorectal cancer. In addition, a series of in vitro and in vivo experiments were performed to demonstrate the role of SIRT2 in tumour angiogenesis. We showed that silencing SIRT2 significantly suppressed tumour angiogenesis. Mechanistically, the knockdown of SIRT2 inhibited STAT3 phosphorylation, causing decreased secretion of VEGFA. Notably, we found that SIRT2 directly interacted with STAT3 and affected the phosphorylation of STAT3 and the translocation of phosphorylated STAT3 to the nucleus. Importantly, a series of rescue experiments suggested that the function of SIRT2 in tumour angiogenesis depends on the STAT3/VEGFA signalling pathway. Our findings provide insight into the important role of SIRT2 in colon tumour angiogenesis and suggest that SIRT2/STAT3/VEGFA might be a novel prognostic biomarker and a potential therapeutic target for patients with colorectal cancer.

## Introduction

Colorectal cancer (CRC) is one of the most common gastrointestinal tract cancers, and its incidence is increasing in most countries^1^. Although many therapeutic strategies, such as surgical resection, targeted molecular drug therapy and combined chemotherapy, have been performed for patients with CRC, the prognosis of CRC is still unfavourable^2–4^.

Accumulating evidence has revealed that angiogenesis, which is a complex process in which new blood vessels arise from pre-existing microvasculature, plays a critical role in tumour development^5,6^. During cancer development, angiogenesis is believed to supply abundant nutrients and oxygen for cancer cell survival^7^. In addition, recent studies have shown that tumour cells strongly promote blood vessel formation by secreting a diversity of angiogenic factors, such as VEGFA, PDGFB, bFGF and EGF^8^. Currently, antiangiogenic therapy has been reported to effectively improve survival rates of patients with CRC, suggesting that inhibition of tumour angiogenesis is a potential way to treat CRC^9–11^. However, antiangiogenic therapy is controversial because patients may have intrinsic or secondary resistance to it^12^. Importantly, the underlying molecular mechanism of the regulation of angiogenesis remains poorly understood. Thus, it is urgent to further explore the mechanisms of tumour angiogenesis to obtain better therapeutic outcomes in patients with CRC.

SIRT2 is a histone deacetylase belonging to the sirtuin family of proteins^13^. Increasing evidence has demonstrated that SIRT2 plays important roles in tumourigenesis and tumour progression. For example, increased expression of SIRT2 is observed in breast cancer, and inhibition of SIRT2 significantly inhibits breast cancer growth^14^. Consistent with this report in breast cancer, a previous study has also shown that knockdown of SIRT2 decreases liver cancer invasion^15^. In colon cancer cell lines, pharmacological inhibition of SIRT2 strongly induces cell cycle arrest^16^. These findings suggest that SIRT2 might act as an oncogene in tumour progression and development. However, in contrast, a previous report has shown that SIRT2-deficient (SIRT2-KO) mice develop certain kinds of tumours through regulation of centrosome amplification^17^. Lower SIRT2 protein expression has been observed in both human head and neck squamous cell carcinoma and oesophageal adenocarcinoma^18,19^. These findings indicate that SIRT2 might function as a tumour suppressor. The above studies clearly show that SIRT2 plays vital roles in tumour progression, such as in the cell cycle and in tumour growth and metastasis, and its roles in tumour development may be dependent on the cellular and tissue context. However, the role of SIRT2 in tumour angiogenesis has not yet been determined.

In this study, we demonstrated that higher expression of SIRT2 occurs in CRC tissues compared with normal samples. Moreover, both in vitro and in vivo functional assays showed that knockdown of SIRT2 significantly decreased angiogenesis. We further showed that the function of SIRT2 in tumour angiogenesis was dependent on the STAT3/VEGFA signalling pathway in CRC cells. Our findings reveal the novel molecular mechanism by which SIRT2 plays a critical role in angiogenesis and provide a potential targeted therapeutic strategy for treating CRC.

## Results

### SIRT2 was overexpressed and related to poor prognosis in CRC

**Fig. 1:**
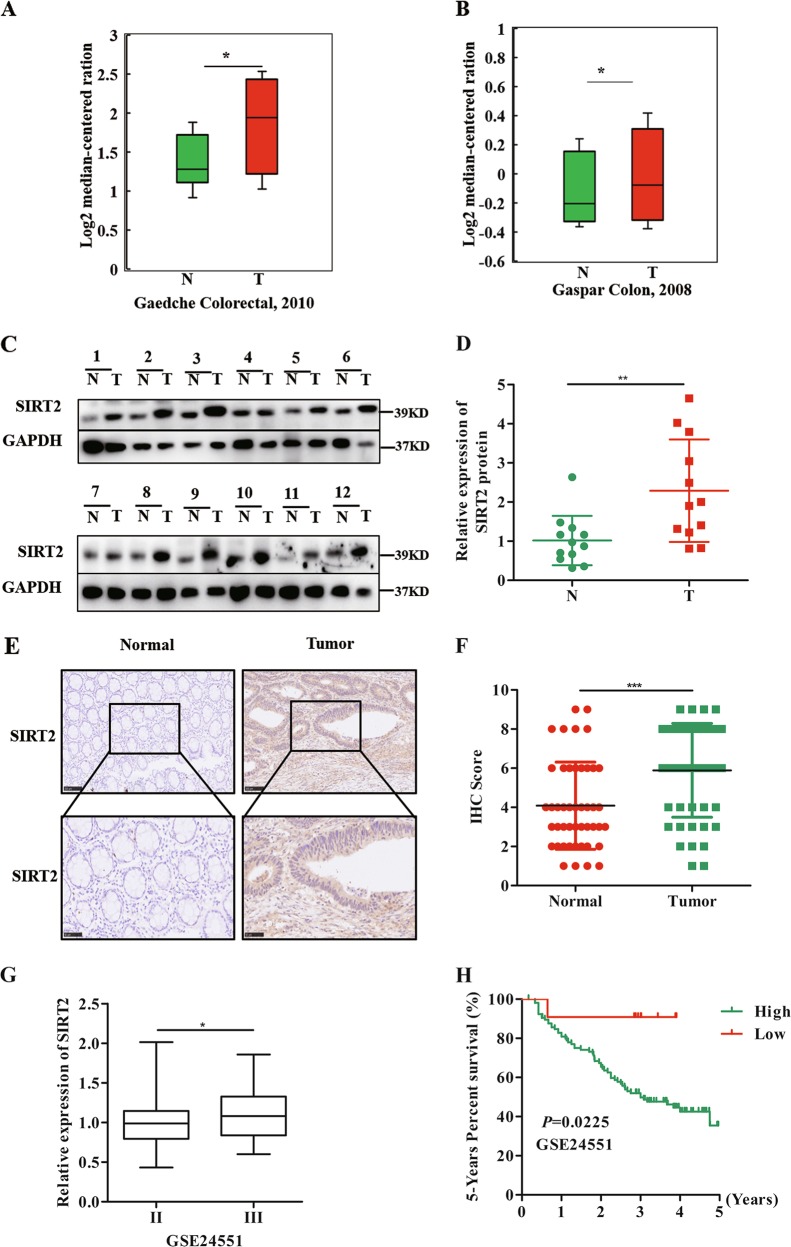
SIRT2 was overexpressed and related to poor prognosis in CRC. **a, b** Analysis of SIRT2 levels in CRC tumour tissues compared to those in normal samples using Oncomine databases. *P < 0.05. **c** Analysis of SIRT2 protein levels in 12 paired CRC samples and adjacent normal tissues. **d** Quantification of the protein levels of SIRT2 in 12 paired CRC samples and adjacent normal tissues. N: normal, T: tumour, **P < 0.01. **e** Immunohistochemistry (IHC) staining of SIRT2 in CRC tumour tissues and adjacent normal tissues (*n* = 46). **f** Vertical scatter plots showing that the IHC score for SIRT2 in the CRC tissue was significantly higher than that in the adjacent normal tissue. ***P < 0.001. **g** SIRT2 levels in different TNM stage patients from GSE24551. *P < 0.05. **h** Kaplan–Meier analysis showing that the high expression of SIRT2 is related to poor prognosis in CRC from GSE24551. *P* = 0.0225

To examine the potential role of SIRT2 in CRC development, we first analysed the expression of SIRT2 in normal samples and CRC tissues with the Oncomine database. As shown in Fig. 1a, b, the level of SIRT2 was higher in CRC tissues compared with normal samples in two different CRC datasets. To confirm this finding, we further examined the protein expression of SIRT2 in our clinical CRC specimens. Consistent with the above results, we found that SIRT2 was highly expressed in CRC tissues (Fig. 1c, d). In addition, we employed IHC assays to assess the expression level of SIRT2 in CRC tissues. As shown in Fig. 1e, f, the IHC results showed that the average level of SIRT2 was higher in CRC tissues than in matched normal samples. These results strongly suggest that SIRT2 is involved in CRC development.

Next, to further investigate the clinical value of SIRT2, we analysed the publicly available CRC dataset GSE24551. As shown in Fig. 1g, SIRT2 upregulation was preferentially correlated with advanced TNM stage. More importantly, patients with high SIRT2 levels had a lower 5-year survival rate (Fig. 1h). In another online database (https://www.proteinatlas.org/), we also found that SIRT2 was upregulated in CRC tissues compared with normal samples and that patients with high-SIRT2 levels had a shorter overall survival time (Supplementary figure 1A, B). Taken together, the above findings indicate that increased SIRT2 expression is related to aggressive CRC development and poor prognosis.

### Inhibition of SIRT2 attenuates tumour-induced angiogenesis

**Fig. 2:**
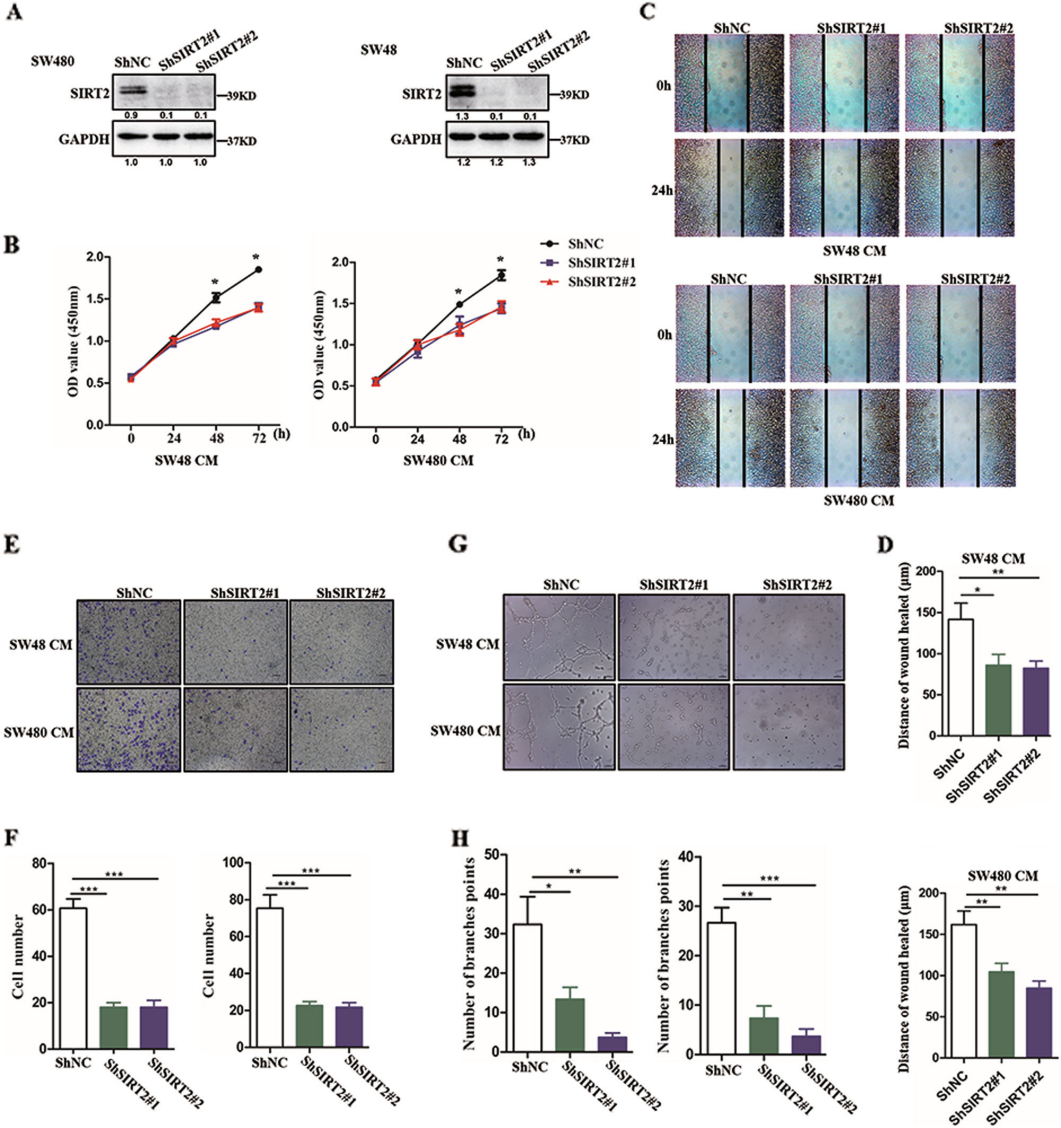
Inhibition of SIRT2 attenuates tumour-induced angiogenesis. **a** Western blot analysis was used to examine the protein levels of SIRT2 in ShNC cells and ShSIRT2 cells. **b** CCK assay showing the proliferative ability of HUVECs incubated with conditional medium (CM) from ShNC cells or ShSIRT2 cells. Each bar represents the mean ± SD of three independent experiments. *P < 0.05, ***P < 0.001. **c**, **d** Wound healing assay showing the migratory ability of HUVECs treated with conditional medium (CM) from ShNC cells or ShSIRT2 cells. The migration distance is shown in the bar graph. Each bar represents the mean ± SD of three independent experiments. *P < 0.05. **e**, **f** Transwell assay showing the migratory ability of HUVECs treated with conditional medium (CM) from ShNC cells or ShSIRT2 cells. The number of migrated cells is shown in the bar graph. Each bar represents the mean ± SD of three independent experiments. *P < 0.05, **P < 0.01. **g**, **h** Effect of CM from ShNC cells or ShSIRT2 cells on tube formation in HUVECs. The number of tubes counted is shown in the bar graph. Each bar represents the mean ± SD of three independent experiments. *P < 0.05

Given the critical role of angiogenesis in tumour progression, we asked whether SIRT2 contributed to tumour-induced angiogenesis. To test this hypothesis, we first established stable shRNA-SIRT2 (ShSIRT2)-expressing SW48 and SW480 cell lines using two lentivirus containing two different ShRNA sequences against SIRT2. Both western blot and qPCR assays showed that shRNA-SIRT2 effectively reduced the protein and mRNA expression levels of SIRT2 in SW480 and SW48 cells (Fig. 2a, Supplementary figure 2A, B). Interestingly, we found that the proliferative ability was significantly attenuated in human umbilical vein endothelial cells (HUVECs) incubated with conditioned medium (CM) from SW480 cells expressing shRNA-SIRT2 (ShSIRT2) compared with that in cells incubated with CM from SW480 cells expressing ShRNA-NC (ShNC) (Fig. 2c). In accordance with this result, attenuated proliferative ability was also observed in HUVECs incubated with CM from ShSIRT2 SW48 cells.

Although cell proliferation plays an important role in angiogenesis, previous studies have revealed that cell migration is also a vital step in angiogenesis. Thus, we further assessed the role of CM from ShSIRT2 cells in HUVEC migration. As shown in Fig. 2c, d, treatment with CM from ShSIRT2 cells strongly inhibited the migratory ability of HUVECs in a wound healing assay. In addition, in order to confirm that this phenotype was caused by ShSIRT2, we used a transwell assay to examine the migratory ability of HUVECs. As expected, the results were similar to those of the wound healing assay and demonstrated that CM from ShSIRT2 cells strongly suppressed HUVEC migration compared with CM from ShNC cells (Fig. 2e, f). Furthermore, a tube formation assay was performed on HUVECs in Matrigel treated with CM from ShNC or ShSIRT2 cells. As shown in Fig. 2g, h, lower tube formation was observed in the ShSIRT2 groups than in the ShNC groups. Taken together, these data strongly indicate that inhibition of SIRT2 abolished tumour-induced angiogenesis.

### VEGFA is required in angiogenesis mediated by SIRT2

**Fig. 3:**
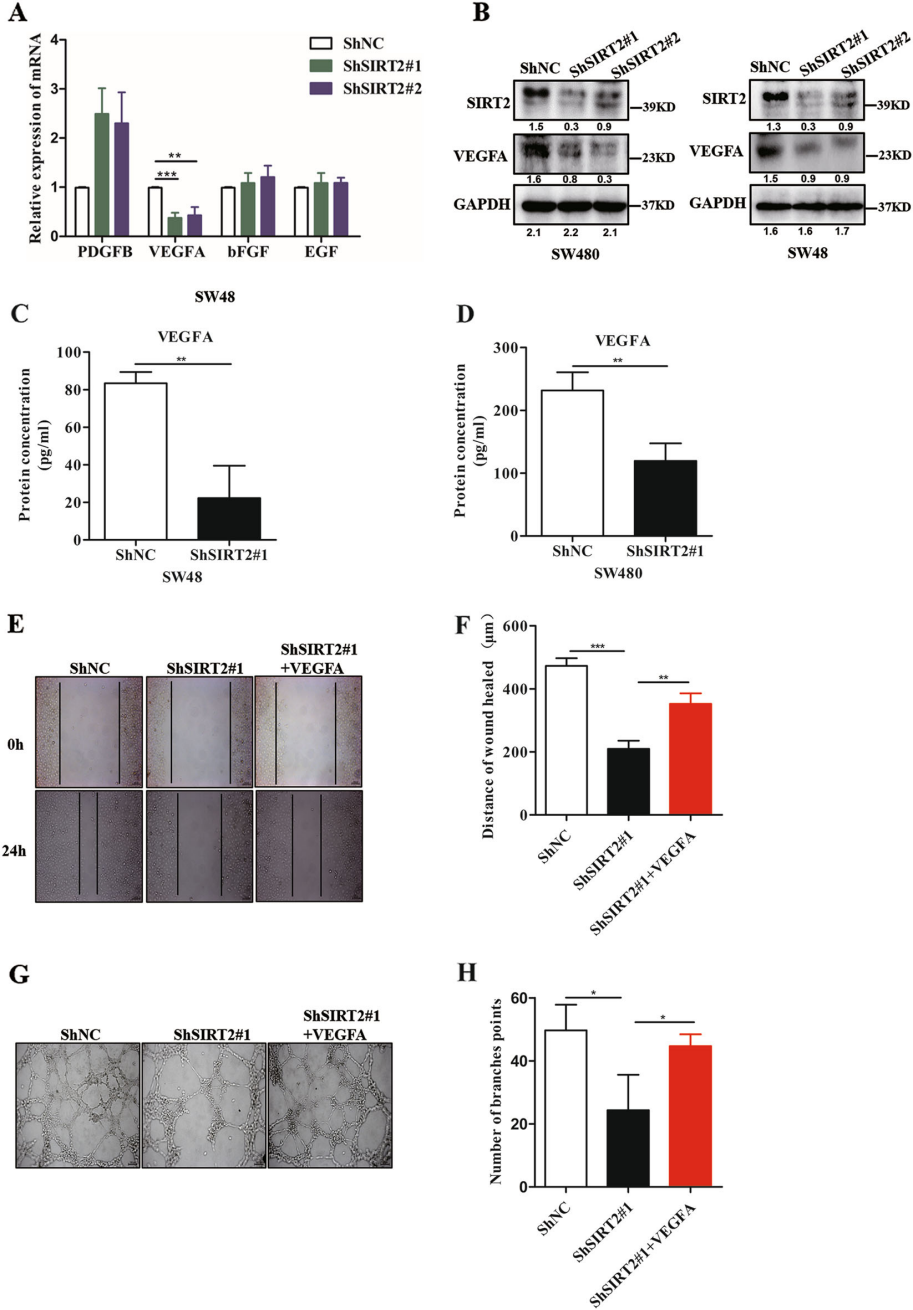
VEGFA is required for angiogenesis mediated by SIRT2. **a** The mRNA levels of the indicated genes in ShNC SW48 cells and ShSIRT2 SW48 cells were examined by qPCR assay. Each bar represents the mean ± SD of three independent experiments. **P < 0.01, ***P < 0.001. **b** Western blot assay showing the VEGFA protein expression in the ShNC and ShSIRT2 CRC cell lines. **c**, **d** VEGFA production was examined by ELISA in the indicated culture supernatants. Each bar represents the mean ± SD of three independent experiments. **P < 0.01. **e**, **f** Wound healing assay showing the migratory ability of HUVECs treated with conditional medium (CM) from ShNC SW480 cells and ShSIRT2 SW480 cells or treated with CM added with VEGFA (100 ng/ml). The migration distance is shown in the bar graph. Each bar represents the mean ± SD of three independent experiments. **P < 0.01, ***P < 0.001. **g**, **h** Effect of CM from ShNC SW480 cells and ShSIRT2 SW480 cells or CM added with VEGFA (100 ng/ml) on tube formation in HUVECs. The number of tubes counted is shown in the bar graph. Each bar represents the mean ± SD of three independent experiments. *P < 0.05

Mounting research has shown that many tumours secrete a diversity of angiogenic factors (such as VEGFA, PDGFB, bFGF and EGF) to promote tumour angiogenesis. Thus, we asked if SIRT2 was involved in the secretion of these angiogenic factors. To test this hypothesis, we first examined the mRNA expression of various kinds of angiogenic factors in ShNC SW48 cells and ShSIRT2 SW48 cells. Interestingly, we found that knockdown of SIRT2 significantly inhibited VEGFA expression, but not the expression of other factors (Fig. 3a). Meanwhile, we examined the other VEGF members upon the shRNA SIRT2 manipulations. As shown in Supplementary figure 3A, VEGFA was the most downregulated gene by knockdown SIRT2, in addition, VEGFC also was downregulated by silenced SIRT2. However, VEGFC was thought to play a major role in lymphangiogenesis but not tumour angiogenesis. Those results strongly indicated that VEGFA may contribute to the effect of SIRT2 on tumour angiogenesis. The western blot results also showed that knockdown of SIRT2 strongly inhibited VEGFA protein expression (Fig. 3b). We next examined the secretion of VEGFA in the culture supernatants from ShNC and ShSIRT2 cells. Consistent with the above results, the ELISA results also demonstrated that the concentration of VEGFA decreased in CM from ShSIRT2 cells compared with that in CM from ShNC cells (Fig. 3c, d).

To investigate the potential role of VEGFA in SIRT2-mediated tumour angiogenesis, we evaluated the migratory ability of HUVECs incubated with CM from ShSIRT2 SW480 cells and stimulated with VEGFA. As shown in Fig. 3e, f and Supplementary figure 4A, we found that the anti-migratory effect of CM from ShSIRT2 cells on HUVECs was blocked by the addition of VEGFA. Furthermore, the antiangiogenic effect of CM from ShSIRT2 cells on HUVECs was strongly reversed by the addition of VEGFA (Fig. 3g, h). These results suggest that the regulation of tumour angiogenesis by SIRT2 depends on the secretion of VEGFA.

### Knockdown of SIRT2 inhibits the activation of the STAT3 signalling pathway in CRC

**Fig. 4:**
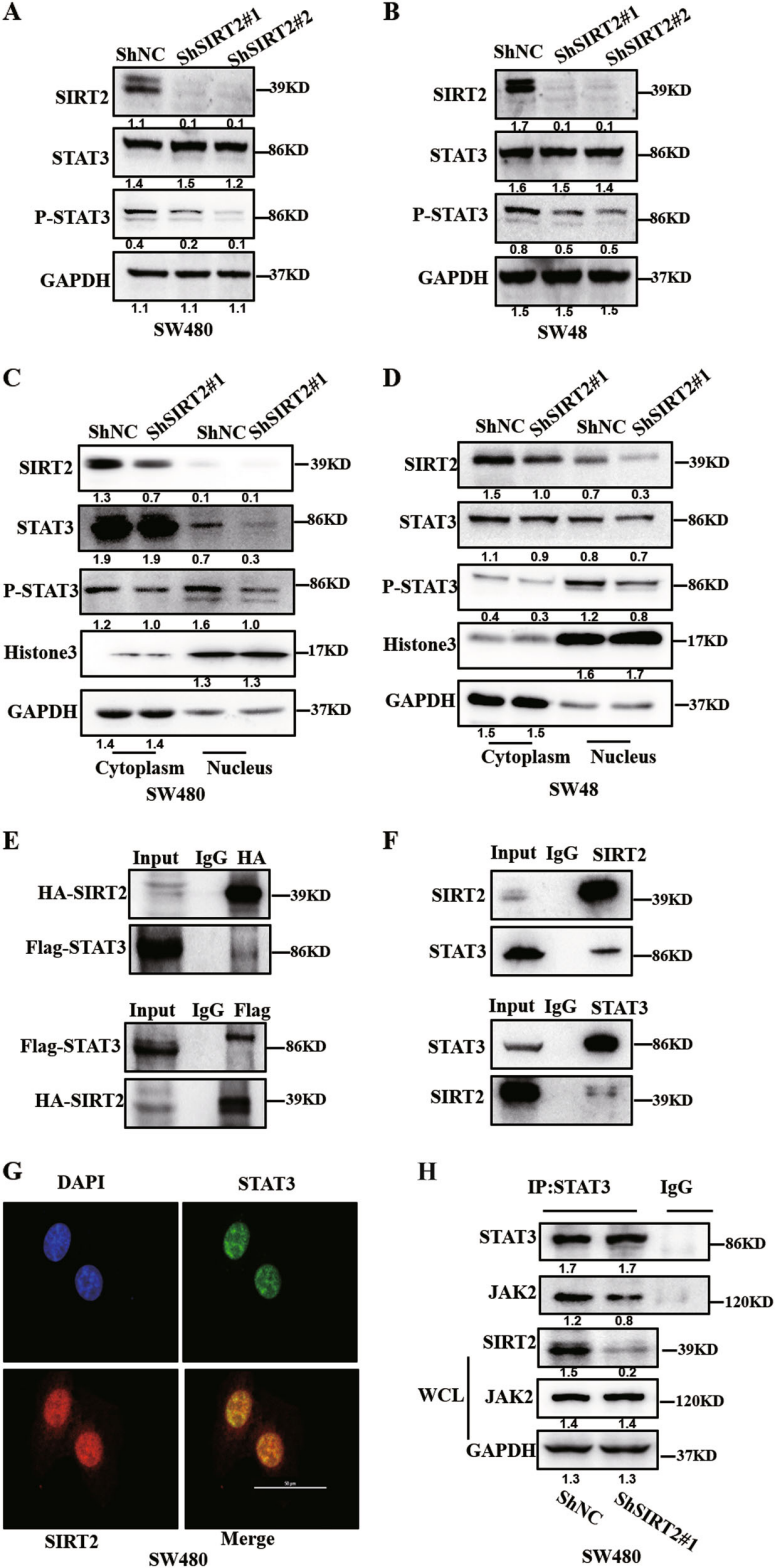
Knockdown of SIRT2 inhibits the activation of the STAT3 signalling pathway in CRC. **a, b** The levels of the indicated proteins were measured by using western blot assay in the indicated cells. GAPDH was the internal reference. **c, d** Western blot assay showing the nuclear total STAT3 and p-STAT3 expression of SW48 and SW480 cells with or without inhibition of SIRT2. **e** 293T cells were cotransfected with FLAG-STAT3 and HA-SIRT2 plasmids, and then the cell lysate was used to perform an immunoprecipitation assay. Western blot showing the interaction between Flag-STAT3 and HA-SIRT2 in 293T cells. **f** SW480 cells were lysed and then the cell lysate was used to perform an immunoprecipitation assay. Western blot showing the endogenous interaction between STAT3 and SIRT2 in SW480 cells. **g** IF staining showing co-localization of SIRT2 (red) and STAT3 (green) in SW480 cells. Scale bar: 50 μm. **h** IP analyses for IgG and STAT3 in ShNC and ShSIRT2#1 SW480 cells followed by western blot analyses with the indicated antibodies

Given that previous reports have shown that a specific inhibitor of SIRT2 abolished the STAT3 signalling pathway^21^ and have demonstrated the important role of the well-known STAT3 pathway in tumour angiogenesis^22,23^, we hypothesized that SIRT2 regulates the secretion of VEGFA via the STAT3 signalling pathway. To test this hypothesis, we examined the phosphorylation and total levels of STAT3 in CRC cell lines. As shown in Fig. 4a, b, the western blot results demonstrated that knockdown of SIRT2 could strongly decrease the phosphorylation of STAT3 and have a slight effect on total STAT3 expression in both SW48 and SW480 cell lines. Given that the phosphorylated STAT3 acts as a transcription factor by translocating to the nucleus to activate genes involved in tumour angiogenesis, we detected whether SIRT2 mediates the phosphorylation of STAT3 in the nucleus. Interestingly, the results demonstrated that in both cell lines, knockdown of SIRT2 significantly reduced the levels of phosphorylated STAT3 in the nucleus (Fig. 4c, d).

To elucidate how SIRT2 regulates the STAT3 pathway, we analysed protein-protein interactions. We co-transfected HEK293T cells with Flag-STAT3 and HA-SIRT2 plasmids. The western blot data showed that Flag-STAT3 co-immunoprecipitated with HA-SIRT2; consistent with this finding, HA-SIRT2 also co-immunoprecipitated with Flag-STAT3 in HEK293T cell lysates (Fig. 4e). In agreement with this result, we observed that endogenous SIRT2 could bind to endogenous STAT3 in SW480 cells by co-immunoprecipitation assays (Fig. 4f). Immunofluorescence assay (IF) also showed that SIRT2 mainly co-located with STAT3 in the nuclei of SW480 cells (Fig. 4g). Many reports have showed that STAT3 could be phosphorylated by receptor-associated Janus kinase 2 (JAK2) in response to stimulation by cytokines and growth factors. Thus, it prompted us to hypothesize that whether SIRT2 could affect JAK2/STAT3 pathway to regulate the phosphorylation of STAT3. As shown in Fig. 4h, we interestingly found that knockdown SIRT2 attenuated the combination of JAK2 and STAT3 by immunoprecipitation assay and the total level of JAK2 protein was not affected by knockdown of SIRT2 in SW480 cells. These data indicated that SIRT2 might be involved in the regulation of the phosphorylation STAT3 via interfering with the interaction between STAT3 and JAK2 in CRC.

### The effect of SIRT2 on angiogenesis depends on the STAT3/VEGFA signalling pathway in CRC

**Fig. 5:**
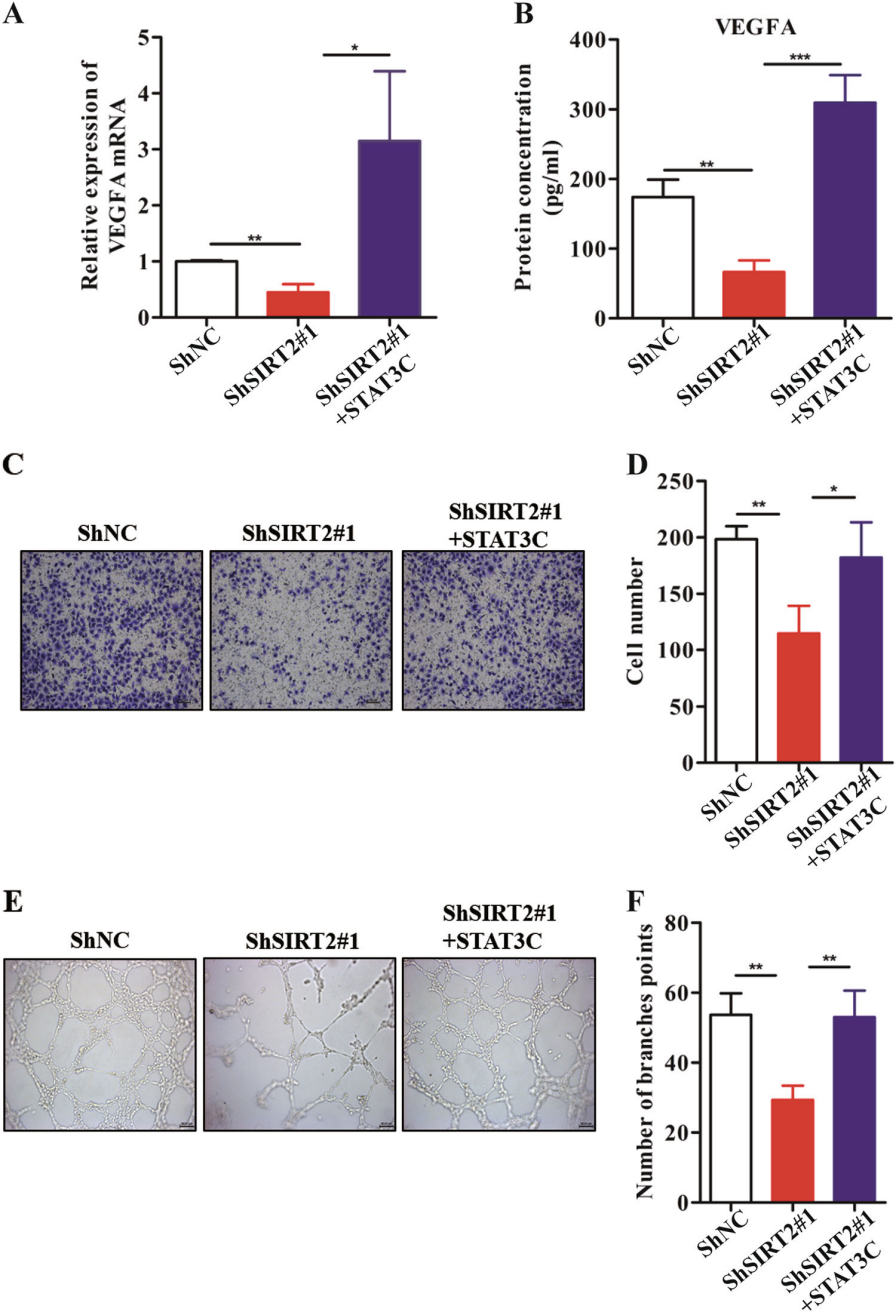
The effect of SIRT2 on angiogenesis depends on the STAT3/VEGFA signalling pathway in CRC. **a** VEGFA mRNA levels were examined by using qPCR assay for the indicated cells (ShNC SW480 cells, ShSIRT2 SW480 cells and ShSIRT2 SW480 cells transfected with STAT3C). Each bar represents the mean ± SD of three independent experiments. *P < 0.05, **P < 0.01. **b** VEGFA production was measured in the culture supernatants. Each bar represents the mean ± SD of three independent experiments. **P < 0.01, ***P < 0.001. **c, d** The migratory ability of HUVECs was assessed by using a transwell assay in the indicated cells. The number of migrated cells is shown in the bar graph. Each bar represents the mean ± SD of three independent experiments. *P < 0.05, **P < 0.01. **e, f** The tube formation of HUVECs treated with different CM was observed, and the number of tubes formed is shown in the bar graph. Each bar represents the mean ± SD of three independent experiments. **P < 0.01

To further examine the role of STAT3 in SIRT2-mediated VEGFA secretion in CRC, we first constructed a constitutively active STAT3 plasmid (STAT3C) according to the methods in a previous report^24^. Next, we transfected ShSIRT2 cells with STAT3C plasmids. Interestingly, we found that the decrease in VEGFA mRNA levels mediated by knockdown of SIRT2 was completely reversed by the overexpression of STAT3C (Fig. 5a). More importantly, the decreased VEGFA secretion mediated by knockdown of SIRT2 was also significantly reversed by the overexpression of STAT3C (Fig. 5b), suggesting that knockdown of SIRT2 reduced VEGFA expression through inhibition of the STAT3 pathway. Furthermore, to test the role of the STAT3 pathway in tumour angiogenesis mediated by SIRT2, we collected CM from each group. As expected, the impaired migratory ability of HUVECs incubated with CM from ShSIRT2 SW480 cells was reversed by CM from ShSIRT2 SW480 cells transfected with STAT3C (Fig. 5c, d). In addition, our results revealed that after treatment with CM from ShSIRT2 SW480 cells transfected with STAT3C, tube formation was accelerated compared to that after treatment with CM from ShSIRT2 SW480 cells (Fig. 5e, f). Taken together, these results strongly indicate that the effect of SIRT2 on angiogenesis depends on the STAT3/VEGFA signalling pathway in CRC.

### Knockdown of SIRT2 suppresses tumour angiogenesis in vivo

**Fig. 6:**
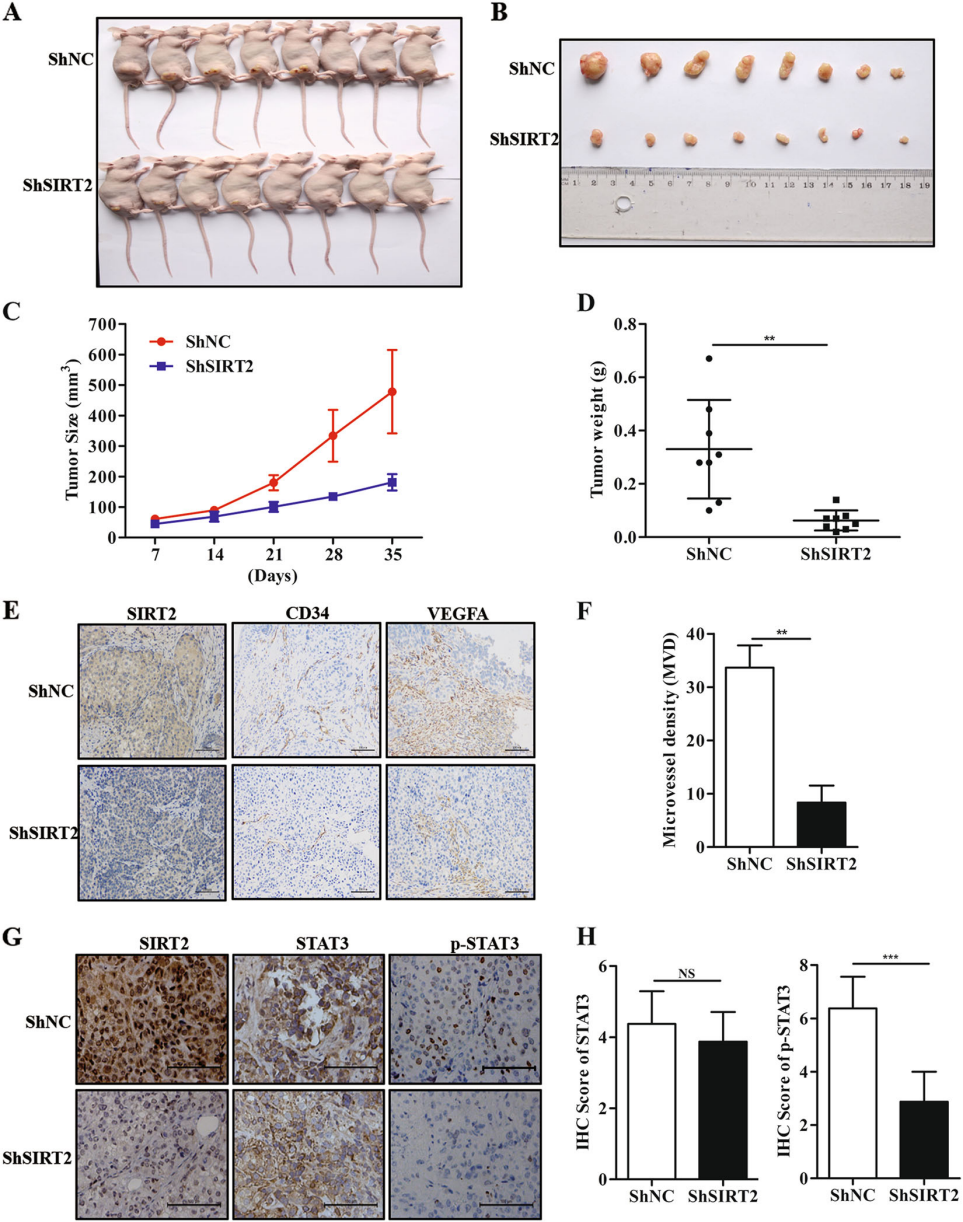
Knockdown of SIRT2 suppresses tumour angiogenesis in vivo. **a, b** Representation of human CRC in xenografted mice. **c** Mean tumour volumes were measured at the indicated time points after injection of ShNC SW480 or ShSIRT2 SW480 cells (*n* = 8). **d** The weights of the tumours from sacrificed mice were examined in the ShNC SW480 cell group and the ShSIRT2 SW480 cell group. **P < 0.01. **e** IHC assay showing the expression of SIRT2, VEGFA and CD34 in the ShNC group and the ShSIRT2 group. Scale bar: 100 μm. **f** The microvascular density of the tumours was determined by quantifying the CD34 staining in the two groups. **P < 0.01. **g** Representative SIRT2, STAT3 and p-STAT3 staining by IHC in ShNC control and ShSIRT2 xenografted tumour tissues. **h** Histograms showed the expression of STAT3 and p-STAT3 in the ShNC group and the ShSIRT2 group. NS: No Significant, ***P < 0.001. Scale bar: 100 μm

To further confirm the role of SIRT2 in tumour angiogenesis, we created a xenograft model in nude mice via the subcutaneous injection of ShNC SW480 cells or ShSIRT2#1 SW480 cells. Thirty-five days after injection, the mice were sacrificed for subsequent experiments. As shown in Fig. 6a–c, the tumours from the mice injected with ShNC SW480 cells were significantly larger than those from the mice injected with ShSIRT2 SW480 cells. In addition, the weight of the tumours from the mice injected with ShNC SW480 cells was significantly greater than that of the tumours from the mice injected with ShSIRT2 SW480 cells (Fig. 6d), suggesting that SIRT2 is involved in CRC development. Of note, we examined the relationship between SIRT2 expression and microvascular density. As expected, we found that the tumours from the ShNC group had a higher expression of CD34 compared with the tumours from the ShSIRT2 group by IHC staining assay (Fig. 6e). Meanwhile, the IHC staining results also showed higher expression of VEGFA in the group with the ShNC SW480 cells compared with that in the group with the ShSIRT2 SW480 cells (Fig. 6e). Furthermore, we quantified the microvessel density (MVD) of the two groups, as shown in Fig. 6f; the tumours with low SIRT2 expression had a lower MVD value compared to that of the control group. Considering the regulation of SIRT2 on the STAT3 signalling pathway, we further evaluated the association of SIRT2 expression with STAT3 and p-STAT3 in xenograft samples. The IHC assay demonstrated that higher expression of p-STAT3 in the group with the ShNC SW480 cells compared with that in the group with the ShSIRT2 SW480 cells, however, there was no change in STAT3 expression between the two groups (Fig. 6g, h). Collectively, these results strongly indicate that knockdown of SIRT2 inhibits CRC angiogenesis and growth through STAT3/VEGFA signalling pathway in vivo.

## Discussion

There is increasing evidence supporting an important role of SIRT2 in tumour development. However, the role of SIRT2 in tumour angiogenesis is still unclear. We hypothesized that a possible link exists between SIRT2 and tumour angiogenesis in CRC. In this study, we found that SIRT2 was upregulated in CRC tissues compared with that in matched normal samples. More importantly, we showed that high expression of SIRT2 was associated with a poor prognosis and a late tumour stage in CRC. The most striking finding of this study was that inhibition of SIRT2 inhibited proliferation, migration and tube formation in vitro. Several results suggested that the role of SIRT2 in tumour angiogenesis is dependent on the STAT3/VEGFA signalling pathway. In different CRC cell lines, we demonstrated that silencing SIRT2 markedly decreased the phosphorylation of STAT3 and suppressed the secretion of VEGFA. Furthermore, the suppression of VEGFA signals caused by knockdown of SIRT2 could be reversed by the overexpression of STAT3C. Our in vitro angiogenesis assay demonstrated that impaired tube formation mediated by knockdown of SIRT2 was abolished by the addition of VEGFA or the overexpression of STAT3C. In addition, our data showed that angiogenesis is significantly impaired in ShSIRT2 SW480 mice.

Previous studies have demonstrated that the role of SIRT2 in tumour progression is dependent on the context of tissue type. In leukaemia cells, SIRT2 promotes cell proliferation via enhancing the production of NADPH^25^. McGlynn LM. et al. demonstrated that SIRT2 is involved in breast cancer and acts as a tumour suppressor or oncogene depending on the breast tumour grade^26^. Furthermore, it has been reported that SIRT2 directly deacetylates Slug to prevent Slug protein degradation and then promotes basal-like breast cancer development. In addition, there is a report showing that SIRT2 can promote liver cancer metastasis by regulating EMT progression. These findings clearly demonstrate that SIRT2 is involved in various aspects of tumour progression. However, its precise role in tumour progression is controversial. To date, there have been no studies investigating whether SIRT2 is involved in tumour angiogenesis. Mounting evidence has shown that tumour angiogenesis is critical for cancer proliferation and metastasis, and strategies based on antiangiogenic treatment have achieved definite success. This evidence prompted us to ask whether SIRT2 regulates tumour angiogenesis in CRC, which is one of the most common cancers worldwide. Interestingly, our results showed that SIRT2 was overexpressed in CRC and might be a prognosis marker. In addition, we reported that knockdown of SIRT2 significantly suppressed tube formation in CRC. These data imply that inactivation of SIRT2 in CRC has the potential to suppress CRC development through the regulation of tumour angiogenesis.

There exists a common molecular mechanism for tumour angiogenesis. A diversity of angiogenic factors (such as VEGFA, PDGFB, bFGF and EGF) secreted from tumour cells have been reported to play an important role in tumour angiogenesis^27^. Of note, many studies have shown that different kinds of cancer exhibit various responses to antiangiogenic treatment due to the abilities of different cancer cells to secrete various angiogenic factors^28–31^. We showed, in this study, that knockdown of SIRT2 inhibited VEGFA mRNA expression, but not PDGFB, bFGF or EGF expression. This finding suggested that the role of SIRT2 in tumour angiogenesis might involve VEGFA in CRC. Mechanistically, VEGFA is in fact the master mediator of tumour angiogenesis in different cancer types. Herein, we further demonstrated that the effect of SIRT2 knockdown on CRC angiogenesis was reversed by stimulation with VEGFA in vitro.

Several upstream transcriptional factors, such as STAT3 and HIF1A, are known to activate VEGFA and promote the secretion of VEGFA in various cancer types^22,23,32–34^. It is worth noting that STAT3 has been reported to be sufficient to stimulate the secretion of VEGFA under normal culture conditions. However, HIF1A functions as a pro-angiogenic factor mainly under hypoxic conditions^35^. Similar to the findings for STAT3, we found in this work that inhibition of SIRT2 significantly attenuated tumour angiogenesis under normal culture conditions. Thus, we examined the changes in the STAT3 signalling pathway in CRC cells with inhibited SIRT2. Our results are consistent with previous results indicating that pharmacological inhibition of SIRT2 markedly decreases the phosphorylation of STAT3; in the present study, knockdown of SIRT2 strongly abolished the phosphorylation of STAT3 and delayed nuclear localization in CRC. In addition, we interestingly found that knockdown SIRT2 attenuated the combination of JAK2 and STAT3 to affect STAT3 signalling pathway. We were interested in exploring whether STAT3 was involved in tumour angiogenesis mediated by SIRT2 in CRC, so we conducted related experiments. The results presented herein clearly showed that continual activation of STAT3 could completely reverse the inhibitory effect of SIRT2 inhibition on tumour angiogenesis, suggesting that the role of SIRT2 in tumour angiogenesis is dependent on the STAT3 signalling pathway. Based on our data, we believe that the physiological or pathological inhibition of SIRT2 will be a novel therapeutic strategy for CRC treatment.

## Materials and methods

### Cell culture and Reagents

SW48, SW480 and HUVECs were all purchased from the American Type Culture Collection (Manassas, VA, USA) and cultured in Dulbecco’s modified Eagle’s medium (Gibco BRL, Grand Island, NY) supplemented with 10% foetal bovine serum (Gibco BRL) and 1% penicillin/streptomycin in a humidified incubator with 5% CO2 at 37 ℃. Antibodies against GAPDH (sc-137179), Flag (sc-166355) and HA (sc-7392) were purchased from Santa Cruz Biotechnology (Santa Cruz, CA, USA). Antibodies against SIRT2 (ab211033) and VEGFA (ab1316) were obtained from Abcam (Cambridge, MA, USA). Antibodies against Histone 3 (#4499), JAK2 (#3230), STAT3 (#9139,) and phospho-Stat3 (Tyr705) (#9145) were purchased from Cell Signaling Technology (Danvers, MA, USA). Human VEGF factors were purchased from Multi Sciences. VEGFA was used to culture HUVEC cells in an amount of 100 ng/ml.

### Stable cell lines and plasmid construction

Lentiviral vectors containing ShNC (negative control, NC) and ShRNA-SIRT2 (SIRT2-specific target sequence: ShSIRT2#1, 5’-GCCATCTTTGAGATCAGCTAT-3’; ShSIRT2#1, 5’-GCTAAGCTGGATGAAAGAGAA-3’) particles were bought from Shanghai Genechem Co., Ltd. The viral supernatant was used to transduce SW48 and SW480 cells according to the manufacturer’s protocol. STAT3C plasmids were generated using a Fast Mutagenesis System (TransGen Biotech, Beijing, China). The primers for STAT3C were 5’-AAGATCATGGATTGTACCTGCATCCTGGTGTCTCCA-3’ and 5’-GCAGGTACAATCCATGATCTTATAGCCCATGATGAT-3’. The full-length SIRT2 was cloned into a pCMV-HA vector. The primers for SIRT2 were 5’-ATGGCAGAGCCAGACCCCTCT-3’ and 5’-TCACTGGGGTTTCTCCCTCTC-3’.

### CRC clinical samples and immunohistochemistry (IHC) assay

CRC samples and matched adjacent normal tissues were obtained from patients with CRC. All patient tissues used in this study were approved by the Ethics Committee of Tongji Hospital, Wuhan, China, and were collected in agreement with the Declaration of Helsinki. Informed consent was obtained from each patient involved in this experiment before this study was carried out. Immunohistochemistry was performed as described in a previous study^20^. SIRT2 staining intensity was categorized: no staining as 0, weak as 1, moderate as 2 and strong as 3. The percentage of cells stained was categorized: no positive cells as 0, less than 25% positive cells as 1, 25%–50% positive cells as 2, 50%–75% positive cells as 3 and more than 75% positive cells as 4. We calculated the score of each sample by multiplying the SIRT2 staining intensity with the percentage scale. For statistical analysis, scores of 0 to 7 were considered low expression and scores of 8 to 12 considered high expression.

### CCK8 assay

HUVECs (2 × 10^3^/100 μl) were seeded into 96-well plates and treated with CM from ShNC SW480 cells or ShSIRT2 SW480 cells. At the indicated times (0 h, 24 h, 48 h and 72 h), 10 μl of CCK was added to the medium. Following incubation for 2 h, the cell proliferation was assessed by measuring the absorbance of the medium.

### Cell migration assay

HUVECs (4 × 10^4^/200 μl) were suspended using medium without serum. Next, cells were seeded into the upper chamber of a transwell chamber, and 500 μl of CM from ShNC cells or ShSIRT2 cells was added to the lower chamber. Then, cells were incubated for 16 h and stained with violet solution. Images were captured with a light microscope.

### Wound healing assay

HUVECs were seeded into 6-well plates. When 98% confluency was reached, the media were replaced with CM from ShNC cells or ShSIRT2 cells, and 200 μl sterile plastic pipette tips were used to scratch the cells. The wound distances were measured using a microscope. After 24 h, the wound healing distances were photographed and measured.

### Tube formation assay

Matrigel (50 μl) was placed into 96-well plates and solidified at 37 °C for 30 min. Then, 3 × 10^4^ HUVECs were separately suspended in CM from ShNC cells or ShSIRT2 cells and seeded into the pre-coated Matrigel wells (BD Biosciences, NJ, USA). After continuing to incubate for 8 h, the tube formation was observed under an inverted microscope (Eclipse model TS100; Nikon, Tokyo, Japan), and the tube formation ability of the HUVECs was assessed by counting the number of branch points.

### Immunofluorescence

SW480 cells were seeded on coverslips cultured in 24-well plates. When the cells were attached to the coverslips, they were fixed with 4% formaldehyde and then washed with cold PBS twice. Next, the cells were permeabilized with 0.1% Triton X-100 for 5 min, blocked with 1% bovine serum albumin for 1 h, and treated with the indicated primary antibodies overnight. After being incubated with secondary antibodies and DAPI, the cells were visualized under a confocal laser scanning microscope (Olympus FLUOVIEW FV1000).

### ELISA

VEGFA levels were measured with ELISA kits according to the manufacturer’s instructions (#EK0539, Boster Biological Technology Co. Ltd, Wuhan, China).

### Western blot and Immunoprecipitation assay

For western blot assays, protein samples from tissues or cells were separated by SDS-PAGE and transferred onto PVDF membranes (Millipore, MO, USA). The bands were incubated with their corresponding primary antibodies at 4 °C overnight and were then incubated with the corresponding HRP-conjugated secondary antibodies for 1 h at room temperature. The bands were visualized with ECL reagents (Thermo Fisher, MA, USA). For immunoprecipitation assays, SW480 cells were cotransfected with Flag-STAT3 and HA-SIRT2 plasmids for 48 h. Then, the cells were harvested and lysed in NP40 lysis buffer for 30 min. The supernatants were precleared with 10 μl of protein A/G agarose beads for 2 h. The Flag or HA primary antibody was added into the supernatants and incubated at 4 °C overnight. After incubation with 50 μl of protein A/G agarose beads for 4 h at 4 °C, the beads were boiled with loading buffer. Then, the protein samples were used for the western blot assay.

### Quantitative Real-time PCR (qPCR)

Total RNA was extracted with TRIzol (Invitrogen, Carlsbad, CA). Then, cDNA was synthesized using PrimeScriptRT Mix (Takara, Dalian, China). Then, the mRNA for the indicated genes was detected with an ABI 7300 Real-time PCR (RT-PCR) System using SYBR Green PCR Master Mix (Takara, Dalian, China). The sequences of the gene-specific primer were as follows: SIRT2: forward: 5’-ATCCACCGGCCTCTATGACAA-3’, reverse: 5’-CGCATGAAGTAGTGACAGATGG-3’; GAPDH: forward: 5’-ATGGGGAAGGTGAAGGTCGG-3’, reverse: 5’-GACGGTGCCATGGAATTTGC-3’; PDGFB: forward: 5’-CTCGATCCGCTCCTTTGATGA-3’, reverse: 5’-CGTTGGTGCGGTCTATGAG-3’; VEGFA: forward: 5’-AGGGCAGAATCATCACGAAGT-3’, reverse: 5’-AGGGTCTCGATTGGATGGCA-3’; bFGF: forward: 5’-AGTGTGTGCTAACCGTTACCT-3’, reverse: 5’-ACTGCCCAGTTCGTTTCAGTG-3’, EGF, forward: 5’-TGTCCACGCAATGTGTCTGAA-3’, reverse: 5’-CATTATCGGGTGAGGAACAACC-3’. VEGFB: forward: 5’- GAGATGTCCCTGGAAGAACACA-3’, reverse: 5’-GAGTGGGATGGGTGATGTCAG-3’; VEGFC: forward: 5’-ATGTGTGTCCGTCTACAGATGT-3’, reverse: 5’-GGAAGTGTGATTGGCAAAACTGA-3’; VEGFD: forward: 5’-TCCCATCGGTCCACTAGGTTT-3’, reverse: 5’-AGGGCTGCACTGAGTTCTTTG-3’; VEGFE: forward: 5’-ATTCACAGCCCAAGGTTTCCT-3’, reverse: 5’-GGGTCTTCAAGCCCAAATCTT-3’ and PGF: forward: 5’-GAACGGCTCGTCAGAGGTG-3’, reverse: 5’-ACAGTGCAGATTCTCATCGCC-3’. GAPDH was used as an internal control.

### Mouse tumour xenograft model

Four-week-old male BALB/c nude mice were purchased from Huafukang Bio-Technology (Beijing, China). Sixteen mice were randomly divided into two groups. Then, the mice were subcutaneously injected with 50 × 10^4^ SW480 cells stably expressing ShNC or ShSIRT2#1. Beginning 7 days after injection, the tumour volume was examined every week. After 4 weeks, the mice were sacrificed for subsequent experiments. All animal experiments were conducted according to the NIH Guide for the Care and Use of Laboratory Animals and were approved by the Institutional Animal Care and Use Committee at Tongji Hospital.

### Statistical analysis

All data were analysed with GraphPad Prism 5.0 (La Jolla, CA, USA). Two-tailed Student’s t-tests and Kaplan-Meier analysis were used when appropriate. P < 0.05 was considered statistically significant.

## Supplementary information

supplemental figure 1

supplemental figure 2

supplemental figure 3

supplemental figure 4
