# Supplementary material for: Inhibition of SIRT2 limits tumour angiogenesis via inactivation of the STAT3/VEGFA signalling pathway
Source: Cell Death Dis. 2018 Dec 18;10(1):9. doi: 10.1038/s41419-018-1260-z (PMC6315023; doi:10.1038/s41419-018-1260-z)
Supplement: Supplementary file 1 — supplemental figure 1 [file 41419_2018_1260_MOESM1_ESM.pdf]

Supplement Figure 1

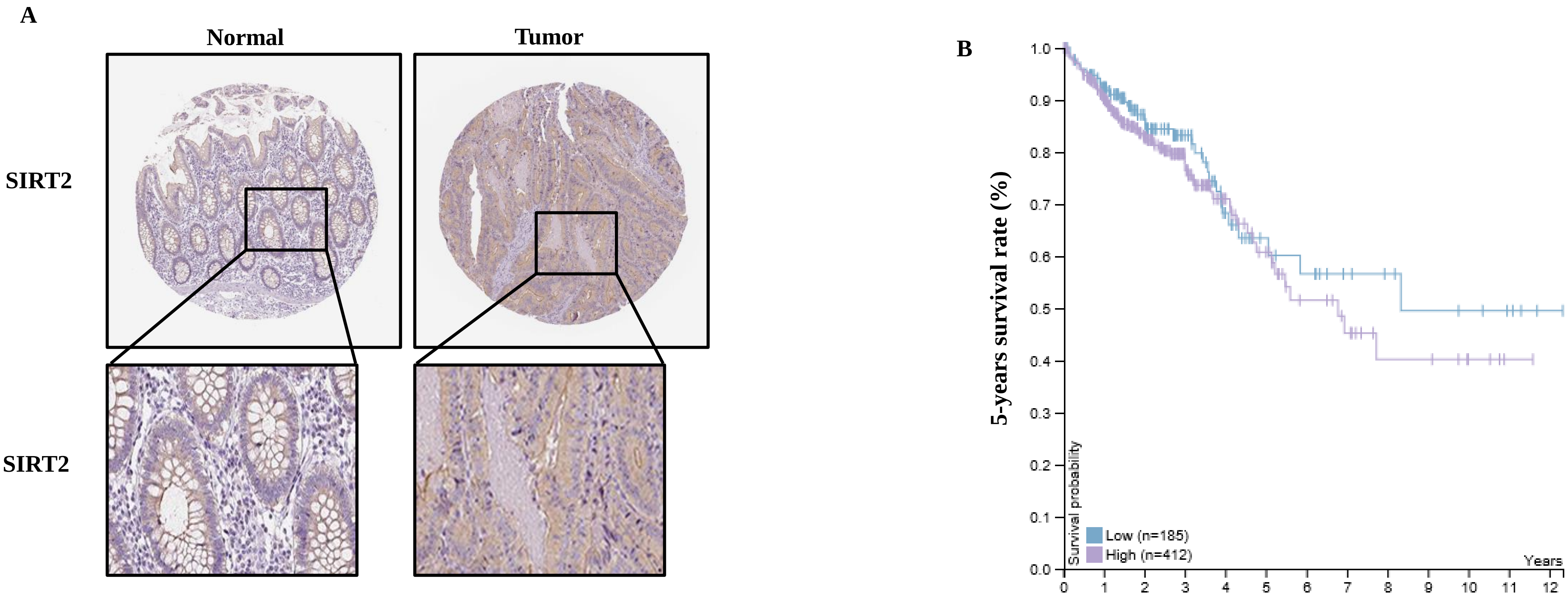

A. Representative immunohistochemical (IHC) staining of SIRT2 in normal tissues and tumor samples from online databases (https://www.proteinatlas.org/); B. Kaplan-Meier's analyses were performed according to SIRT2 protein expression on colorectal cancer patients from online databases (https://www.proteinatlas.org/).
