# Supplementary material for: Inhibition of SIRT2 limits tumour angiogenesis via inactivation of the STAT3/VEGFA signalling pathway
Source: Cell Death Dis. 2018 Dec 18;10(1):9. doi: 10.1038/s41419-018-1260-z (PMC6315023; doi:10.1038/s41419-018-1260-z)
Supplement: Supplementary file 2 — supplemental figure 2 [file 41419_2018_1260_MOESM2_ESM.pdf]

Supplement Figure 2

A

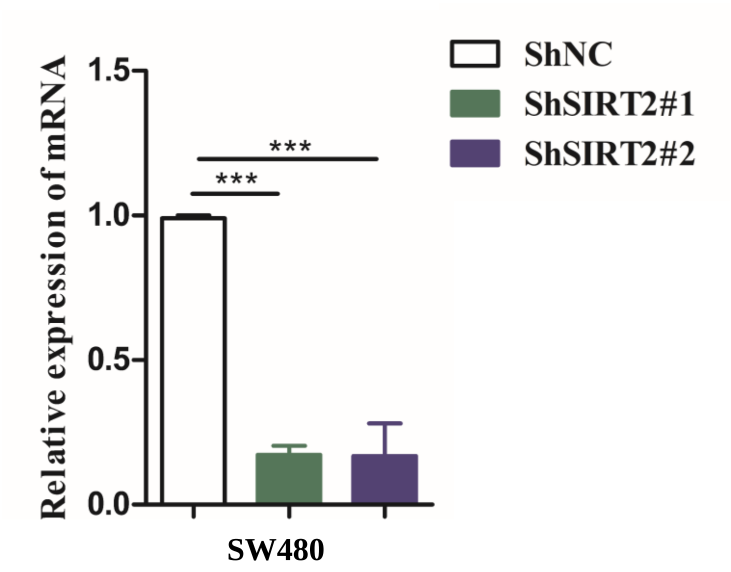

B

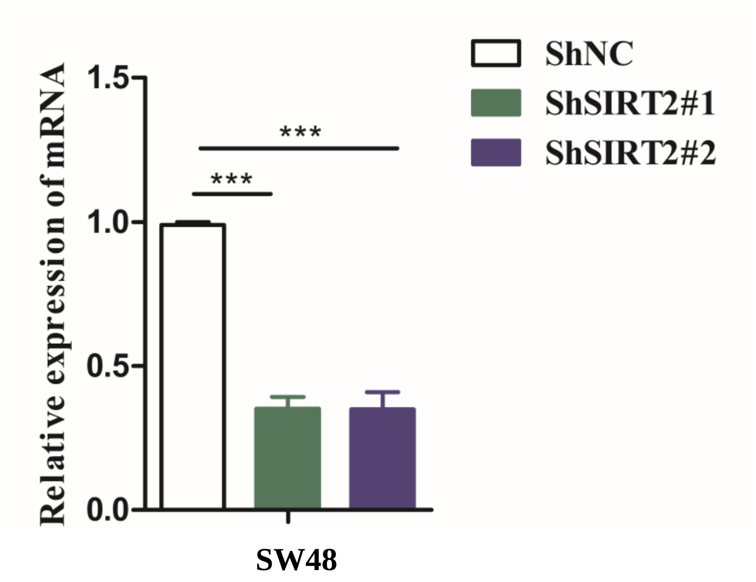

A. The mRNA level of SIRT2 was detected by quantitative PCR between SW480 ShNC cells and SW480 ShSIRT2 cells, ***P<0.001; B. The mRNA level of SIRT2 was detected by quantitative PCR between SW48 ShNC cells and SW48 ShSIRT2 cells, ***P<0.001.
