# Supplementary material for: Inhibition of SIRT2 limits tumour angiogenesis via inactivation of the STAT3/VEGFA signalling pathway
Source: Cell Death Dis. 2018 Dec 18;10(1):9. doi: 10.1038/s41419-018-1260-z (PMC6315023; doi:10.1038/s41419-018-1260-z)
Supplement: Supplementary file 3 — supplemental figure 3 [file 41419_2018_1260_MOESM3_ESM.pdf]

Supplement Figure 3

A

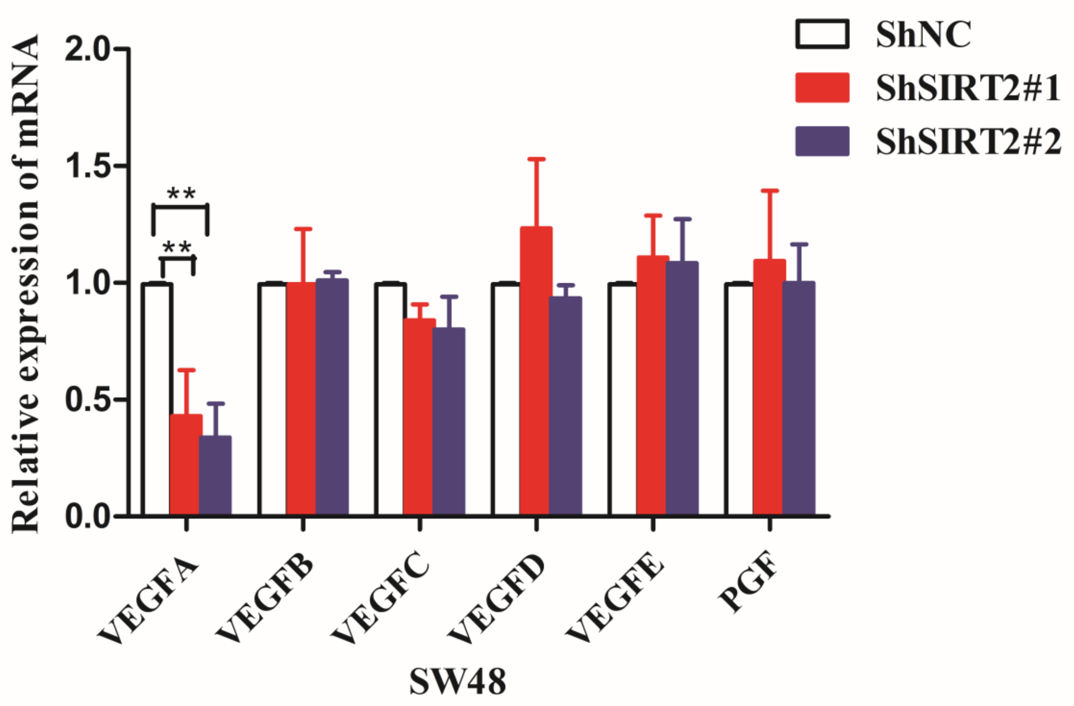

A. The mRNA level of indicated gene was detected by quantitative PCR between SW48 ShNC cells and SW48 ShSIRT2 cells, **P<0.01.
