# Supplementary material for: Inhibition of SIRT2 limits tumour angiogenesis via inactivation of the STAT3/VEGFA signalling pathway
Source: Cell Death Dis. 2018 Dec 18;10(1):9. doi: 10.1038/s41419-018-1260-z (PMC6315023; doi:10.1038/s41419-018-1260-z)
Supplement: Supplementary file 4 — supplemental figure 4 [file 41419_2018_1260_MOESM4_ESM.pdf]

Supplement Figure 4

A

ShNC | ShSIRT2#1 | ShSIRT2#1 +VEGFA

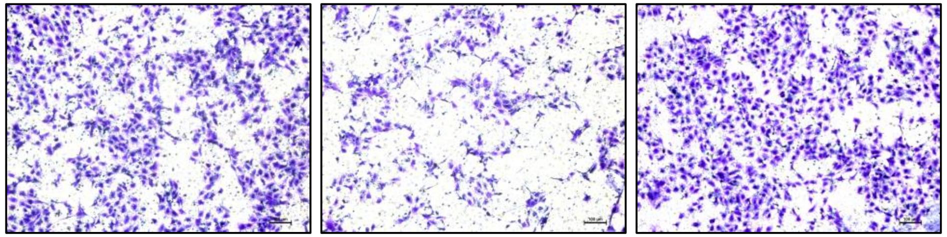

A. Transwell assay showing that the migration ability of HUVECs treated with conditional medium (CM) from ShNC SW480 cells and ShSIRT2#1 SW480 cells or CM added with VEGFA.
